# Gut hormones profile after an Ivor Lewis gastro-esophagectomy and its relationship to delayed gastric emptying

**DOI:** 10.1093/dote/doac008

**Published:** 2022-03-08

**Authors:** Ji Chung Tham, Dimitri J Pournaras, Bruno Alcocer, Rosie Forbes, Arun V Ariyarathenam, Martyn L Humphreys, Richard G Berrisford, Tim J Wheatley, David Chan, Grant Sanders, Stephen J Lewis

**Affiliations:** Peninsula Oesophago-Gastric Centre, University Hospital Plymouth, Plymouth, UK; Department of Upper Gastrointestinal Surgery, North Bristol NHS Trust, Bristol, UK; Peninsula Oesophago-Gastric Centre, University Hospital Plymouth, Plymouth, UK; Peninsula Oesophago-Gastric Centre, University Hospital Plymouth, Plymouth, UK; Peninsula Oesophago-Gastric Centre, University Hospital Plymouth, Plymouth, UK; Peninsula Oesophago-Gastric Centre, University Hospital Plymouth, Plymouth, UK; Peninsula Oesophago-Gastric Centre, University Hospital Plymouth, Plymouth, UK; Peninsula Oesophago-Gastric Centre, University Hospital Plymouth, Plymouth, UK; Peninsula Oesophago-Gastric Centre, University Hospital Plymouth, Plymouth, UK; Peninsula Oesophago-Gastric Centre, University Hospital Plymouth, Plymouth, UK; Department of Gastroenterology, University Hospital Plymouth, Plymouth, UK

**Keywords:** gastric emptying, Ivor Lewis gastro-esophagectomy, esophageal and gastric surgery, esophageal cancer

## Abstract

Delayed gastric emptying (DGE) is common after an Ivor Lewis gastro-esophagectomy (ILGO). The risk of a dilated conduit is the much-feared anastomotic leak. Therefore, prompt management of DGE is required. However, the pathophysiology of DGE is unclear. We proposed that post-ILGO patients with/without DGE have different gut hormone profiles (GHP). Consecutive patients undergoing an ILGO from 1 December 2017 to 31 November 2019 were recruited. Blood sampling was conducted on either day 4, 5, or 6 with baseline sample taken prior to a 193-kcal meal and after every 30 minutes for 2 hours. If patients received pyloric dilatation, a repeat profile was performed post-dilatation and were designated as had DGE. Analyses were conducted on the following groups: patient without dilatation (non-dilated) versus dilatation (dilated); and pre-dilatation versus post-dilatation. Gut hormone profiles analyzed were glucagon-like peptide-1 (GLP-1) and peptide tyrosine tyrosine (PYY) using radioimmunoassay. Of 65 patients, 24 (36.9%) had dilatation and 41 (63.1%) did not. For the non-dilated and dilated groups, there were no differences in day 4, 5, or 6 GLP-1 (*P* = 0.499) (95% confidence interval for non-dilated [2822.64, 4416.40] and dilated [2519.91, 3162.32]). However, PYY levels were raised in the non-dilated group (*P* = 0.021) (95% confidence interval for non-dilated [1620.38, 3005.75] and dilated [821.53, 1606.18]). Additionally, after pyloric dilatation, paired analysis showed no differences in GLP-1, but PYY levels were different at all time points and had an exaggerated post-prandial response. We conclude that DGE is associated with an obtunded PYY response. However, the exact nature of the association is not yet established.

## INTRODUCTION

The incidence of delayed gastric emptying (DGE) is between 10 and 50%^1–3^ after an Ivor Lewis gastro-esophagectomy (ILGO). Patients with post-operative DGE appear to have an increased risk of an anastomotic leak.^4^ Therefore, understanding the pathophysiology of DGE after an ILGO is important.

Currently, some surgeons advocate a pyloric drainage procedure, mechanically or chemically, to prevent DGE.^5–8^ The fundamental rationale for pyloric intervention was that the vagotomized stomach would have an increased pyloric tone post-operatively, and hence, predisposing patients to DGE. However, there is inconsistency in the efficacy of pyloric drainage procedures in preventing DGE,^1–4^^,^^9^ placing doubt in the etiology of DGE.

The pathophysiology of DGE after an ILGO is complex and likely to be multifactorial. Potential mechanisms may include denervation, external influences from the central nervous system, and hormonal and myogenic changes.^10^ An area that is not well understood is the association between gut hormone profile (GHP) changes and the development of DGE. It has been established that gut hormones, such as glucagon-like peptide-1 (GLP-1) and peptide tyrosine tyrosine (PYY), are increased after an ILGO and are associated with gastrointestinal symptoms, such as early satiety, gastrointestinal pain or discomfort, altered taste, and diarrhea.^11^^,^^12^ Briefly, GLP-1 is an incretin that is secreted by cells in the intestine, which, in normal physiology, also increases gastric emptying time, promotes satiety, and results in weight loss.^13–15^ PYY reduces gastric secretion and is also secreted in the intestine with the concentration of the hormone increasing toward the distal intestine.^16–18^ It was then concluded in a recent study that GLP-1 was not associated with any changes to conduit emptying or intestinal transit times.^19^ However, all patients in that study had undergone a pyloroplasty peri-operatively. Hence, further characterization of GHP in patients without pyloroplasty is required.

We proposed that the gut hormone response is different in patients who require pyloric dilatation compared to those that do not. The results will allow better understanding of DGE pathophysiology.

## METHODS

Consecutive patients undergoing an ILGO for esophageal cancer between 1 December 2017 and 31 November 2019 were recruited at a single high-volume tertiary esophago-gastric center. All patients with operable esophageal cancer who were physiologically fit for surgery were included. Patients were excluded if they refused surgery, were found to have unresectable cancer during surgery, or refused to participate in the study. Patients were also excluded if they developed complications that may cause symptoms of DGE (paraconduit hernia) during their in-patient stay. Patient demographics and data included were age, gender, body mass index (BMI), American Society of Anaesthesiology (ASA) grade, smoking status, diabetes status, conduit size (width and length), and DGE status. DGE was diagnosed using an algorithm based on chest X-rays and/or NG input/output volumes—measured from day 4 onward, after initiating the patient onto free fluids and with the spigotted NG aspirated every 4 hours.^3^ From the day of the operation, patients were allowed up to 100 mL of water per hour and free fluids from day 3 with the aim for pureed foods at day 5.^3^ The patients with DGE were designated as ‘dilated’ and those with no DGE were designated as ‘non-dilated’. The designation was chosen based on the requirement for balloon dilatation. For patients treated with pyloric dilatation, further analyses as paired groups: ‘pre-dilatation’ and ‘post-dilatation’ based on their intervention timing were conducted. All patients were followed-up for 6 weeks to review for DGE signs and symptoms (DES). DES included nausea, vomiting, early satiety, dysphagia, post-prandial abdominal pain, and radiological evidence of a distended gastric conduit.

All recruited patients had GHPs measured between day 4 and 6 post-operatively at 08:00 h. They were fasted 6 hours prior to and for the entire period of the test. A 100-mL semisolid ice-cream test meal with 193 kcal was given after an initial baseline blood sample test taken in an ethylenediaminetetraacetic acid tube. The ice cream used was from a locally source company and had 17 g carbohydrate, 12 g fat, and 3 g protein. Repeated sampling was conducted for every 30 minutes up to 2 hours. Each sample was immediately spun at 1500 rpm for 10 minutes at 4°C. Plasma was extracted and stored at −80°C.

Radioimmunoassay was used in the analysis for both total GLP-1 and PYY.^14^^,^^16^ Both GLP-1 and PYY analyses were prepared using chloramine T method and were measured using antibodies raised in gut hormone-immunized rabbits.^14^^,^^16^ Endoscopic pyloric balloon (30-mm Rigiflex™ balloon from Boston Scientific®) was used for pyloric dilatation of patients deemed to have DGE. This intervention was only performed from day 7 onward. All patients treated with pyloric dilatation underwent the GHP test again, as described above, on the following day with the same fasting and testing regimen.^3^

Statistical analyses were performed using IBM®’s SPSS® Statistics software version 25 (https://www.ibm.com/analytics/spss-statistics-software). Graphs were drawn using GraphPad Prism™ version 9 (https://www.graphpad.com). Sample size was determined using a DGE incidence of 17.5%^3^ and an estimated reduction of DGE to 0% post-intervention,^20^ power of 80%, *P* = 0.05, and an enrolment ratio of 3:1 (due to feasibility of recruiting adequate numbers for the intervention in a 2-year period). Hence, the required sample size for the DGE group was 20 patients.

Statistical analysis of nominal data was performed using the χ^2^ square test and Fisher’s exact test. All continuous data were non-parametrically distributed. Therefore, univariate analysis between groups and time points were analyzed using the Mann–Whitney U test and the Wilcoxon signed-rank test. The trapezoid rule was used to calculate the area under the curve (AUC) for GHP. All data were presented as median, range, and/or 95% confidence interval (95% CI). A *P*-value of <0.05 was deemed to be statistically significant.

The London Bromley Research Ethics Committee approved the study (REC 17/LO/1759), which was conducted in accordance with the principles of the Declaration of Helsinki with written informed consent provided by all patients.

## RESULTS

**Fig. 1:**
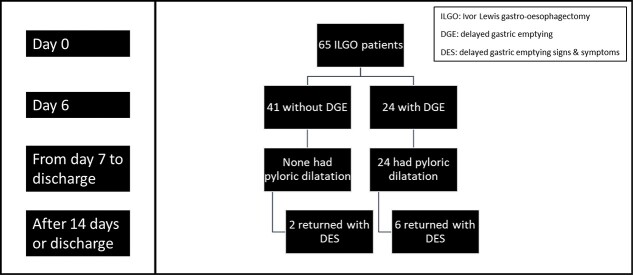
Flow chart of patient progress through the study.

A total of 65 patients were eligible and were included during the 24-month recruitment period. The Consolidated Standards of Reporting Trials flow diagram, as shown in the supplemental data, it described the patient recruitment phases and progress for the study. Figure 1 shows the flow chart for patient progress through the study.

Patient demographics and characteristics are shown in Table 1. There were no differences between the two groups apart from gender. Overall, 24 (36.9%) patients had dilatation and 41 (63.1%) patients did not have dilatation. There were 16 (24.6%) female patients and 49 (75.4%) male patients. The median age of all patients was 70 (43–86) years. The overall median BMI was 26.0 kg/m^2^ (19.0–36.7).

**Table 1:** Characteristics of ‘non-dilated’ and ‘dilated’ patients

	Non-dilated patients, *n* = 41	Dilated patients, *n* = 24	*P*-value
Age	73.10 (44–86)	70.23 (50–81)	0.187
Gender			0.015
Female	6	10	
Male	35	14	
BMI	26.00 (19.00–36.70)	26.00 (19.00–31.50)	0.663
ASA grade			0.088
2	26	20	
3	15	4	
Smoking			0.732
No	12	9	
Ex-smoker	26	14	
Yes	3	1	
Diabetes			0.7331
No	36	19	
Yes	6	4	
Conduit width	5 (4–20)	5 (4–15)	0.563
Conduit length	15 (4–23)	14 (5–24)	0.082

*P*-value < 0.05 was deemed as statistically significant. Parametric data were described as median (range).

**Fig. 2:**
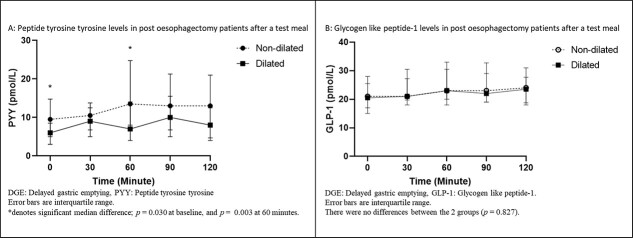
GHP in ‘dilated’ and ‘non-dilated’ groups before and after a test meal.

**Fig. 3:**
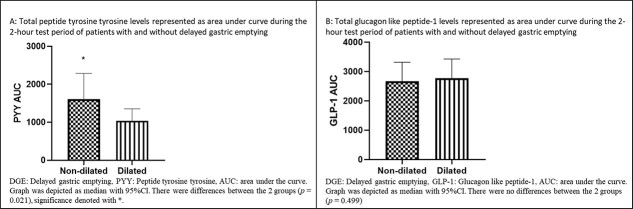
AUC of GHP in ‘dilated’ and ‘non-dilated’ groups before and after a test meal.

Analyses of GHP in patients were grouped as dilated and non-dilated, as shown in Figure 2. For PYY, the AUC for the test period was increased in non-dilated patients (*P* = 0.021) (95% CI for non-dilated [1620.38, 3005.75] relative to dilated [821.53, 1606.18]) (Fig. 3A). For PYY plasma levels, there were differences between the two groups at baseline (non-dilated: 9.50 ρmol/L [95% CI: 5.00, 14.75 ρmol/L]; dilated: 6.00 ρmol/L, [3.00, 8.50 ρmol/L]) (*P* = 0.030); and also, at 60 minutes (non-dilated: 13.50 ρmol/L 8.00, 24.75 ρmol/L; dilated: 7.00 ρmol/L, 4.00, 14.00 ρmol/L) (*P* = 0.003) (Fig. 2A). There were no differences in AUC for GLP-1 for the test duration between the two groups (*P* = 0.499) (95% CI for non-dilated [2822.64, 4416.40 ρmol/L] relative to dilated [2519.91, 3162.32 ρmol/L]) (Fig. 3B). Additionally, there were no differences in the GLP-1 plasma levels between DGE and non-DGE patients for each sampling time point.

**Fig. 4:**
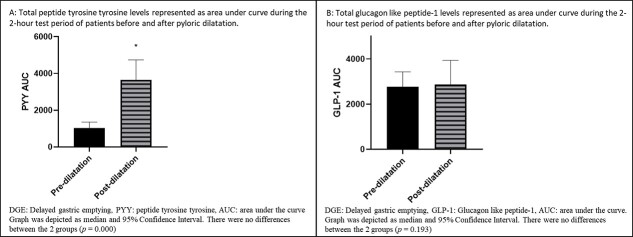
GHP represented as AUC during the 2-hour test period of patients before and after dilatation.

**Fig. 5:**
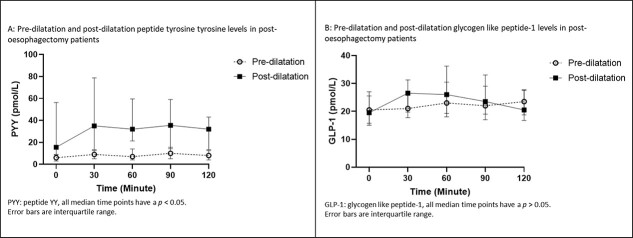
GHP in ‘pre-dilatation’ and ‘post-dilatation’ groups before and after a test meal.

For paired analysis of patients who had undergone pyloric dilatation, the differences between the AUC of PYY for the 2-hour period between the groups was significant (*P* = 0.000), as shown in Figure 4A, whereby the post-dilatation group had higher levels. Paired analyses of the PYY levels were different for all time periods (Baseline *P* = 0.001, 30-minute *P* = 0.001, 60-minute *P* = 0.002, 90-minute *P* = 0.000, and 120-minute *P* = 0.001), as shown in Figure 5A, with the post-dilatation group having had higher levels. An exaggerated peaked response to a test meal can be seen in the post-dilatation group and that response was absent in the pre-dilatation group. Median peaked PYY level was 10 ρmol/L (2.00–36.00 ρmol/L) at 90 minutes for the pre-dilatation group. For the post-dilatation group, median peak level was 34.00 ρmol/L (1.00–165.00 ρmol/L) and it occurred at 30 minutes.

The AUC of GLP-1 showed no differences for the test period (Fig. 4B). The AUC during the test period for GLP-1 was not significant (*P* = 0.193), as shown in Figure 5B. Median peak GLP-1 level occurred at 120 minutes in pre-dilatation patients and was 23.50 ρmol/L (15.00–43.00 ρmol/L), while the median peak level for the post-dilatation group of 29.00 ρmol/L (10.00–98.00 ρmol/L) occurred at 60 minutes.

Overall, all readmissions of patients with DES occurred after 6 weeks. There were eight DES patients. Out of those patients, four (50%) had an anatomical cause (three paraconduit hernia and one ‘folded’ conduit) and four had true DGE. Of the four with anatomical issues, three had DGE as an in-patient and had previously undergone pyloric dilatation. Additionally, of the other four with true DGE, three previously had pyloric dilatation as an in-patient.

From a different perspective and referring to Figure 1, it can be noted that 30% (6/20) who had in-patient pyloric dilatation returned with DES. There were three patients with anatomical issues, while the other three had true DGE in that DES group of patients. For the two DES patients who did not have an in-patient dilatation, one had an anatomical cause while the other had true DGE (Fig. 1). The anatomical cause for that one patient in that non-dilated DES group was a folded conduit at 3 months (Fig. 1).

## DISCUSSION

The results of this study revealed novel information about PYY levels in esophagectomy patients. After an ILGO, the GHP of patients who received pyloric dilatation were different to those that did not have any pyloric intervention, particularly PYY levels. Raised PYY levels in patients with an unaltered gastrointestinal tract occurs secondary to the presence of unimpaired movement of nutrients into the small bowel.^16^ Therefore, the initial conjecture would be to assume that the changes to PYY levels in DGE patients were due to the reduced movement of chyme into the small bowel. Results from this study appeared to show that interventions on the pylorus after an ILGO altered the GHP of patients. For patients who received pyloric dilatation, PYY levels were dampened before dilatation but appeared to be restored after balloon dilatation of the pylorus. Analysis from this study also showed that the differences in PYY levels between non-dilated and dilated patient were also prevalent on AUC analysis. These findings further substantiate the fact that the reduction in the rate of flow of nutrients into the small bowel resulted in attenuated PYY levels. However, if the PYY profile was simply due to the restoration of food presence into the small bowel, then GLP-1 profile should have responded similarly too. Additionally, baseline PYY levels were significantly raised post-dilatation even in the absence of food stimuli. The implications of the differences in baseline levels and GHP profile remain unclear.

The analysis of GHP in ILGO patients revealed no post-prandial changes in GLP-1 levels. The subgroup AUC analysis of GLP-1 patients showed a slight increase post-dilatation, but the results were not significant. Those GLP-1 findings were different compared to findings by Elliott *et al*.^12^ However, compared to the patient group by Elliott *et al*., none of our patients received a pyloroplasty during their ILGO, there was different meal stimulus, and GHP time point measurements were different. Those factors could be the possible explanation for the difference in GHP results. Another possible explanation for the GLP-1 result in this study can be attributable to the fact that changes in GLP-1 levels are smaller compared to PYY and thus, a larger sample size may be required. Other possible reasons for differences in GHP results may be due to sampling variations and/or difference in radioimmunoassay used.

Current literature suggests that GLP-1 may help regulate gut function in the post-ILGO setting,^21^^,^^22^ but the exaggerated post-prandial post-operative GLP-1 level was not associated with the changes in gastric emptying times in ILGO patients, as proposed by Murphy *et al*.^19^ Those findings contradict a review on the physiological effects of GLP-1 on gastric emptying in healthy individuals by Baggio *et al*.^13^ Therefore, it remains uncertain whether modulation of gastric function can be attributed to altered GLP-1 levels after an ILGO. With current evidence for GHP, it can be postulated that DGE after an ILGO may be purely a mechanical issue, and manipulation of gut hormones will not resolve the issue. Additionally, a standardized method of pyloric intervention for DGE in ILGO is required and should be the focus of future research.

With regard to patients who returned with DES, they can be classed as having late DGE. The diagnostic criteria and symptom grading has been determined by a Delphi consensus,^23^ but the underlying cause is still poorly understood and studied. Of the patients who returned with DES in this study, 50% had an anatomical issue to explain their symptoms, and surgical intervention could be employed to fix the mechanical problem. However, the exact cause of late DGE in the other half of patients had not been determined. Therefore, it is uncertain regarding the long-term outcomes of surgical interventions, such as pyloric interventions, for these group of patients.

One of the limitations of this study was the possibility of an inadequate sample size resulting in a non-significant finding in the GLP-1 profile as described above. However, the patient sample from this study was substantially higher than other studies that assessed GHP in esophagectomy patients.^11^^,^^12^^,^^19^^,^^21^ Second, no preoperative GHP baseline test was performed. We did not design this study to include preoperative testing as it was not pragmatically and logistically possible to perform testing in our cohort of patients, as our unit covered a large geographical area. Therefore, preoperative tests for a large number of patients were impractical. Additionally, we decided that an additional preoperative test would have added additional/unnecessary burden and stress to those patients. Another limitation was this study only showed an association of PYY response in DGE, but no conclusion of causation or mechanism can be deduced. Again, further research into the mechanism of DGE is required to show causality. Additionally, we cannot yet explain the underlying reason for a preponderance for dilatation in female patients. Finally, for the analysis of the pre- and post-dilatation groups, there was no control group tested at the same time as the post-dilatation group. Therefore, the possibility of an exaggerated gut hormone response due to a longer fasting time (continual fasting for pyloric dilatation and then fasting for GHP test the following day) in the post-dilatation group could have occurred. However, such phenomenon should not be isolated to PYY only and should also be seen with GLP-1 too.

## CONCLUSION

This study shows that patients who develop DGE have an associated obtunded post-prandial PYY response in the post-operative period. The PYY response appeared to be restored after pyloric dilatation. Unexpectedly, GLP-1 levels remained unchanged after pyloric disruption. Further comprehension of the mechanism of action of gut hormones on gastric function is required to complete the knowledge regarding DGE.

## Supplementary Material

Supplemental_data_doac008

## Notes

Financial support: The study was funded by Plymouth Hospitals Charitable Fund.

Specific author contributions: Conception, design, and data collection: Dr Stephen J. Lewis, Mr Ji Chung Tham, Mr Arun V. Ariyarathenam, Mr LM Humphreys, Mr RG Berrisford, Mr TJ Wheatley, and Mr G Sanders; Interpretation of data: Dr Stephen J. Lewis, Mr Ji Chung Tham, Mr Arun V. Ariyarathenam, Mr Martyn L. Humphreys, Mr Richard G. Berrisford, Mr Tim J. Wheatley, Mr Grant Sanders, Mr Dimitri J. Pournaras, and Dr Stephen J. Lewis; Data collection and reviewing of the manuscript: Mr Bruno Alcocer and Mrs Rosie Forbes; Analysis of the data: Mr Dimitri J. Pournaras, Mr Ji Chung Tham, and Dr Stephen J. Lewis; Drafting and revising manuscript: Mr Ji Chung Tham; Final approval to the submitted article and revised versions: all authors.
