# Supplementary material for: Gut hormones profile after an Ivor Lewis gastro-esophagectomy and its relationship to delayed gastric emptying
Source: Dis Esophagus. 2022 Mar 8;35(10):doac008. doi: 10.1093/dote/doac008 (PMC9742676; doi:10.1093/dote/doac008)
Supplement: Supplemental_data_doac008 [file supplemental_data_doac008.docx]

Consolidated Standards of Reporting Trials (CONSORT) flow diagram for study recruitment

DGE: Delayed gastric emptying
